# Importance of family history of diabetes in computing a diabetes risk score in Korean prediabetic population

**DOI:** 10.1038/s41598-018-34411-w

**Published:** 2018-10-29

**Authors:** Morena Ustulin, Sang Youl Rhee, Suk Chon, Kyu Keung Ahn, Ji Eun Lim, Bermseok Oh, Sung-Hoon Kim, Sei Hyun Baik, Yongsoo Park, Moon Suk Nam, Kwan Woo Lee, Young Seol Kim, Jeong-Taek Woo

**Affiliations:** 1 Department of Medicine, Graduate School, Kyung Hee University, Seoul, Korea; 2 Department of Endocrinology and Metabolism, Kyung Hee University School of Medicine, Seoul, Korea; 3 Department of Biochemistry and Molecular Biology, Kyung Hee University School of Medicine, Seoul, Korea; 4 Division of Endocrinology and Metabolism, Department of Medicine, Cheil General Hospital and Women’s Healthcare Center, College of Medicine, Dankook University, Seoul, Korea; 5 Division of Endocrinology and Metabolism, Department of Internal Medicine, College of Medicine, Korea University, Seoul, Korea; 6 Department of Internal Medicine, College of Medicine, Hanyang University, Guri, Korea; 7 Department of Internal Medicine, College of Medicine, Inha University, Incheon, Korea; 8 Department of Endocrinology and Metabolism, College of Medicine, Ajou University, Suwon, Korea

## Abstract

Prediabetic subjects represent a vulnerable population, requiring special care to reduce the risk of diabetes onset. We developed and validated a diabetes risk score for prediabetic subjects using the Korea National Diabetes Program (KNDP) cohort. Subjects included in the multicenter and prospective cohort (n = 1162) had high diabetes risk at baseline (2005) and were followed until 2012. Survival analysis was performed to analyze the prospective cohort over time, and the bootstrap method was used to validate our model. We confirmed our findings in an external cohort. A diabetes risk score was calculated and the cut-off defined using a receiver operating characteristic curve. Age, body mass index, total cholesterol, and family history of diabetes were associated with diabetes. The model performed well after correction for optimism (C_adj_ = 0.735). A risk score was defined with a cut-off of ≥5 that maximized sensitivity (72%) and specificity (62%), with an area under the curve of 0.73. Prediabetic subjects with a family history of diabetes had a higher probability of diabetes (risk score = 5) irrespective of other variables; this result was confirmed in the external cohort. Hence, prediabetic subjects with a family history of diabetes have a higher probability of developing diabetes, regardless of other clinical factors.

## Introduction

Prediabetes, a condition defined by blood glucose levels that are above the normal range but below diabetes thresholds, is increasing worldwide, and it is estimated that more than 470 million people will have prediabetes by 2030^1^. Approximately 5–10% of prediabetic subjects progress to the diabetic condition every year^2^, underscoring the importance of control measures in this subgroup of subjects to prevent the transition to the diabetic condition.

Diabetes has also increased over the years to become a worldwide problem: the International Diabetes Federation estimated that there were 415 million adults with diabetes worldwide in 2015^3^. If this growing trend is not arrested, it is estimated that there will be 642 million people with diabetes by 2040^3^.

The numbers of Koreans with diabetes and impaired glucose tolerance were 3.3 million and 3.4 million, respectively, in 2010, and are expected to reach 4.3 million and 4.4 million, respectively, in 2030^4^. Diabetes is associated with many comorbidities such as hypertension, coronary artery disease, retinopathy, and nephropathy^5,6^. These complications have become major causes of mortality and morbidity in Korea, as well as a source of increasing healthcare costs, and patients with both microvascular and macrovascular complications have 3.1-fold greater healthcare costs than those without complications^7^. There is also evidence of an association of prediabetes with early forms of nephropathy, diabetic retinopathy, chronic kidney disease, and increased risk of macrovascular diseases^8–10^.

Therefore, prediabetic subjects represent a vulnerable population that requires advanced cares to reduce diabetes onset and to decrease the risk of other complications. Establishing a policy to screen and manage diabetes at a national level for this subgroup is important in terms of prevention. Several risk score systems have been introduced to predict and manage diabetes risk in various countries^11–14^. However, a diabetes risk score system has not yet been applied to the prediabetic population in Korea.

Therefore, we defined and validated a diabetes risk score by analyzing the clinical characteristics of a prospective multicenter cohort of prediabetic Korean subjects. We also assessed this diabetic risk score system in an external cohort to extend our results to the general population.

## Research Design and Methods

### Korea National Diabetes Program (KNDP) cohort

The KNDP cohort is a prospective, multicenter, observational cohort of Korean type 2 diabetes patients and patients at high risk for diabetes. The type 2 diabetes cohort was composed of subjects who satisfied the diagnostic criteria set by the American Diabetes Association in 2004 and who were at least 20 years old^15^. The high-risk group was identified as subjects with impaired glucose tolerance and impaired fasting glucose^15^.

The KNDP cohort study was performed at the following 13 research hospitals: Kyung Hee University Hospital, Kyung Hee University Hospital at Gangdong, Korea University Guro Hospital, Ajou University Hospital, Inha University Hospital, Hanyang University Guri Hospital, Gachon Medical School Gil Hospital, Pusan National University Hospital, Kwandong University Cheil General Hospital and Women’s Healthcare Center, Yeungnam University Medical Center, Inje University Sanggye Baik Hospital, Hallym University Gangdong Sacred Heart Hospital, and the Catholic University of Korea Uijeongbu St. Mary’s Hospital.

The clinical data on the KNDP cohort included medical histories of the subjects, physical examinations, laboratory tests, and surveys. All data related to this cohort were collected in the KNDP written case records, and they were entered, maintained, and managed with active internet-based electronic case report forms (eCRF, http://www.kndp.or.kr) at each of the participating research institutions.

### Subject selection

**Figure 1:**
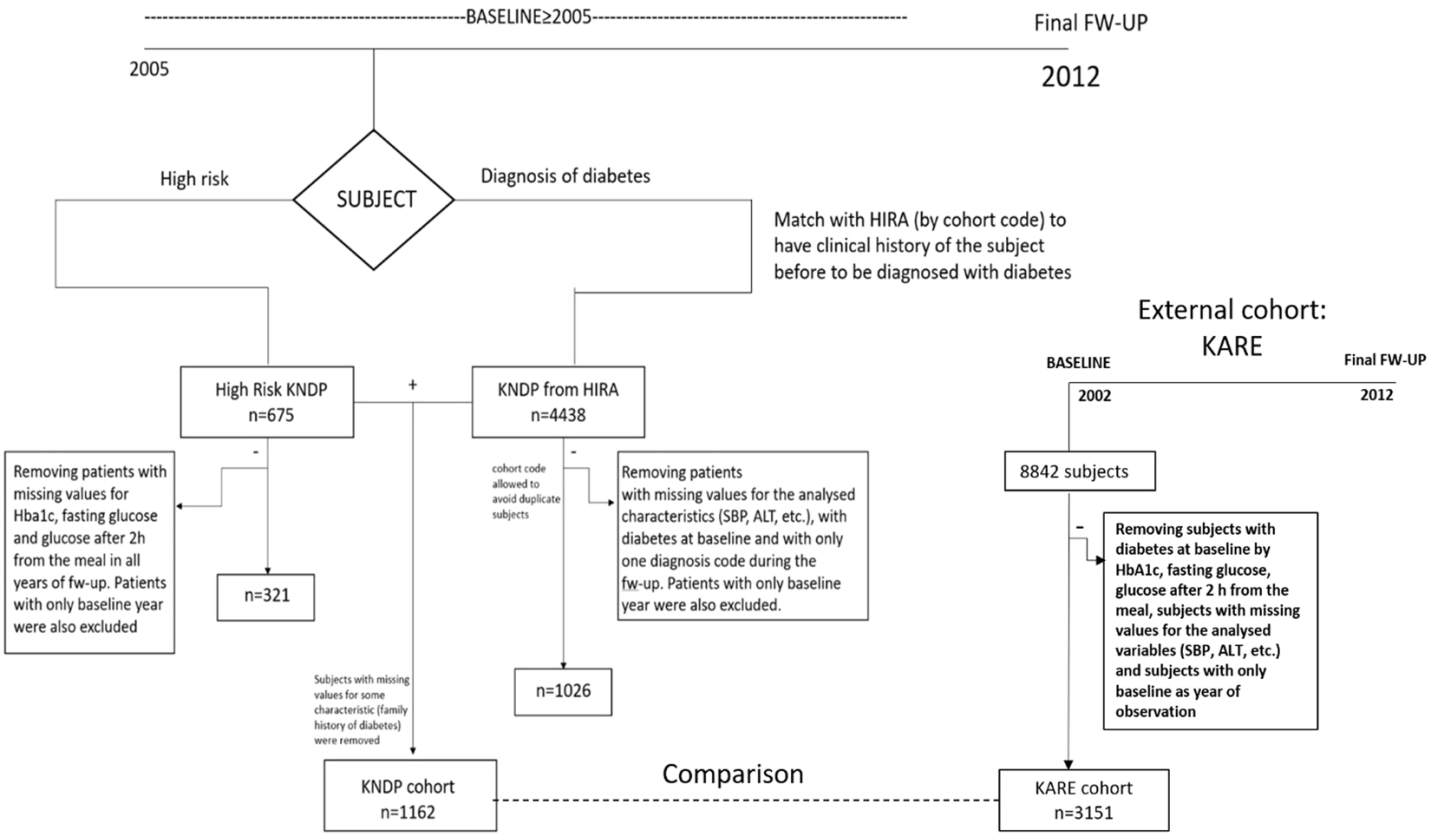
Overview of data sampling methodology.

The KNDP cohort was composed of two groups of subjects: one consisting of people at the prediabetes level according to World Health Organization criteria^16^ (fasting glucose: 100–125 mg/dL; glucose 2 h after eating: 140–199 mg/dL) and the other of subjects with a diagnosis of diabetes during cohort enrollment. In the first group (n = 675), we excluded those who could not be classified correctly regarding diabetes diagnosis due to missing glucose and glycated hemoglobin (HbA1c) values in all years of follow-up. Also, subjects with only baseline information were excluded from the analysis (Fig. 1).

Regarding the second group, we extracted the clinical histories of these subjects prior to diabetes diagnosis from the Health Insurance Review and Assessment Service (HIRA) and followed them in a prospective manner. Subjects with a diagnosis of diabetes during the KNDP enrollment were also present in the HIRA cohort, and subjects were matched between the two cohorts using the unique cohort code for each subject. It was thus possible to obtain past clinical information on these subjects prior to diabetes diagnosis because the HIRA cohort was developed before the KNDP cohort.

HIRA is a governmental organization that reviews National Health Insurance claims for reimbursement. The HIRA database contains claims from a mean of 46 million patients annually, which accounts for 90% of the total Korean population. The data are organized into five sets, including sociodemographic data, diagnostic information, outpatient descriptions, data on healthcare services provided, and information on healthcare service providers^17^.

Therefore, because the HIRA database (n = 4,438) was developed before the KNDP cohort, it was possible to obtain prospective data for this group of subjects and to follow them in an observational study to determine if they presented with diabetes during the follow-up years covered in the HIRA dataset. People with missing values for the analyzed characteristics (systolic blood pressure, total cholesterol, etc.), who presented only once with the diagnosis of diabetes during follow-up were discarded from the analysis (Fig. 1). In this way, using diagnostic codes of the Korean Standard Classification of Disease Version 5 (codes: E11.X, E12.X, E13.X, and E14.X), it was possible to confirm the diabetes diagnoses for this group of subjects during follow-up.

Finally, we collected information on subjects with a high risk of diabetes (n = 321) and on people derived from the group of subjects with type 2 diabetes (n = 1,026). The information on these subjects was put together to compose the KNDP cohort through the cohort codes that exclusively identified subjects, avoiding redundant information (i.e., a subject in the high-risk cohort should not also be present in the HIRA database). Finally, we removed subjects that were missing values for the analyzed characteristics (family history of diabetes), obtaining a total of 1,162 subjects to form the final cohort (Fig. 1).

Because the subjects in the HIRA database potentially had information from 2004–2014 and those in the KNDP cohort from 2005–2012, we followed all 1,162 patients for 7 years (baseline = 2005 and end of follow-up = 2012). Therefore, all patients in the KNDP cohort were free from diabetes at baseline but showed a high risk of diabetes, and were either classified at the prediabetes level or received a diagnosis of diabetes during cohort enrollment.

For the external cohort, we analyzed data from the Korea Association Resource (KARE), which collected information on 8,842 subjects for 10 years (from 2002 until 2012) with health check-ups every 2 years. In this cohort, we selected high-risk subjects at baseline by prediabetic levels of HbA1c, fasting glucose, and glucose 2 h after eating according to the ranges defined by American Diabetes Association^18^. Among these subjects, we removed people who used anti-diabetic drugs at baseline, those who only had information at baseline, and those who were missing values for the variables assessed at baseline: body mass index (BMI), age, systolic blood pressure (SBP), diastolic blood pressure (DBP), gender, family history of diabetes, total cholesterol, alanine transaminase (ALT), aspartate transaminase (AST), triglycerides, high-density lipoprotein (HDL) cholesterol, smoking status, and alcohol consumption. In total, we analyzed 3,151 prediabetic subjects in the KARE cohort (Fig. 1).

### Data and measurements

Variables in this study are based on national health screening data provided by the Ministry of Health and Welfare in Korea, and therefore, they are sufficient for computing a diabetes risk score representative of the Korean population as a whole.

We analyzed several pieces of information for each subject at baseline: demographic data (age and gender), family medical history (close relatives), and lifestyle data such as smoking, alcohol consumption, and physical activity. Laboratory parameters were measured in the morning after overnight fasting, including total cholesterol, fasting glucose, ALT, and AST. We also included information such as BMI, SBP, and DBP in our analysis.

Smoking status was represented by three options: current smoker, never smoker, and ex-smoker. Using this information, we classified subjects into two categories based on whether the subject was a current smoker at the time of the survey. Regarding alcohol consumption, because we did not have information on the quantity of alcohol consumed but only on the frequency (number of days per week), we defined this variable as non-drinkers or drinkers. Physical activity was assessed by weekly duration and frequency. In our analysis, this variable was categorized into two levels that identified whether the subject engaged in regular exercise (duration ≥ 30 min) at least once per week.

All analyzed variables were measured at the baseline and were categorized in order to calculate the diabetes risk score (see Table 1).

**Table 1:** Study participant characteristics.

Variable	n (%) or mean [95% CI]
**1162 patients observed for 7 years (2005–2012)**	
Age (years)	52.51 [51.89, 53.12]
BMI (kg/m^2^)	24.80 [24.61, 25]
Gender (Males)	560 (48%)
SBP (mmHg)	125.09 [124.18, 126.02]
DBP (mmHg)	78.02 [77.43, 78.62]
Fasting glucose (mg/dL)	121.47 [119.02, 123.93]
Total cholesterol (mg/dL)	197.63 [194.99, 200.26]
AST (IU/L)	26.77 [25.99, 27.55]
ALT (IU/L)	30.34 [28.93, 31.75]
Family history of diabetes (yes)	428 (37%)
Current smoker (yes)	161(14%)
Alcohol drinker (yes)	322 (28%)
Physical activity (yes)	370 (32%)
**Categorized variables for computing risk score**	
**Age**	
Age ≥ 53	539 (46%)
**BMI**	
Level 0: BMI < 23	302 (26%)
Level 1: 23 ≤ BMI < 25	253 (22%)
Level 2: BMI ≥ 25	607 (52%)
**SBP**	
Level 0: SBP < 120	387 (33%)
Level 1: 120 ≤ SBP < 140	565 (49%)
Level 2: SBP ≥ 140	210 (18%)
**DBP**	
DBP ≥ 80	598 (51%)
**AST**	
AST > 40	111 (10%)
**ALT**	
ALT > 40	233 (20%)
**Total cholesterol**	
Total cholesterol ≥ 200	516 (44%)

Regarding the KARE cohort, the same variables analyzed in the KNDP cohort were extracted at baseline, with the addition of triglyceride and HDL cholesterol levels, which were not evaluated in the KNDP cohort.

For outcome definition, KNDP subjects from the high-risk group were classified as having diabetes during follow-up if they showed at least one of the following values: fasting glucose ≥126 mg/dL, glucose 2 h after eating ≥ 200 mg/dL, and HbA1c ≥ 6.5. For KNDP subjects with type 2 diabetes, nine questions regarding diabetes status were assessed (consumption of anti-diabetes drugs, previous diagnosis of diabetes) in the HIRA database to ascertain the clinical history of the patient prior to diabetes diagnosis. For these subjects, a confirmation of diagnosis was required to classify the patient with diabetes through the diagnostic codes of the Korean Standard Classification of Disease Version 5. Therefore, subjects who presented with diabetes only once during follow-up were not considered, so that only patients with confirmed diagnoses were included among those classified with diabetes during the study.

Regarding outcome definition in the KARE cohort, we applied the same methods used for the subjects in the high-risk group of the KNDP cohort.

### Statistical analysis

Characteristics of the subjects with high diabetes risk were measured at baseline and expressed as means and 95% confidence intervals (CIs) in cases of continuous variables and as percentages for categorical variables. In order to detect the clinical characteristics affecting diabetes risk, we performed univariate Cox proportional hazards models. Then, using only the variables shown to be significant at α = 5%, we performed a multivariate Cox proportional hazards model applying a stepwise procedure to select the final model. We intentionally retained categorical variables in the models to easily define the risk score.

To validate this model, we performed a bootstrap method, extracting 1,000 samples of the same size as the original data set (n = 1,162). To assess the performance of our model, we computed an overall c-index as defined by Pencina and d’Agostino^19^ and validated it using Harrell’s algorithm for estimating optimism^20^. In this algorithm, 1,000 samples were extracted with replacement from the original dataset, and for each sample, a multivariate model was constructed using the same procedure applied to the original dataset, and a c-index was estimated. Then, for each bootstrap model, we computed its discrimination in the original dataset, fixing the estimated coefficients in each bootstrap model. This procedure allowed us to define the optimism and correct the overall c-index of the original sample.

To compute a risk score from the validated model, we assigned a score for each level of the selected variables: age, BMI, total cholesterol, and family history of diabetes. This score was computed by dividing each coefficient estimate by the minimum value among all coefficients and multiplying the obtained result by a constant (k = 2). In this way, we assigned a score to each variable, taking their effects into account using the coefficient estimates. Proper normalization of all effects can be achieved by dividing each coefficient by the minimum value; indeed, this method enabled us to determine how much stronger a given effect was than the minimum effect. These values were also multiplied by a constant to create a larger range for the risk score. We chose k = 2 to maximize the probability of correctly classifying a subject with diabetes using this risk score. Finally, to define a cut-off for our risk score, we constructed a receiver operating characteristic (ROC) curve computed on all patients that could be classified correctly by diabetes status at the end of the follow-up period (2012). Therefore, the better threshold was that which maximized sensitivity and specificity. Finally, this risk score was validated among the bootstrapped samples and compared with the risk score calculated in the KARE cohort with the same methodology used for the KNDP cohort.

All analyses were performed using SAS 9.4 software (SAS Institute, Cary, NC, USA).

### Ethics Statement

All participants in the study provided informed written consent *a priori* to participation and received a full explanation of the study procedures from relevant medical staff.

All methods were carried out in accordance with the relevant guidelines and regulations, and all experimental protocols were approved by the Institutional Review Board of Kyung Hee University Hospital (KMC IRB 0526-04, KHSIRB-17–091[EA]).

## Results

### Characteristics of the patients at baseline

Over the seven years of follow-up (2005–2012), 69% of patients presented with diabetes during a mean follow-up of 3.97 ± 0.06 years. The incidence of diabetes was 205 cases per 1,000 person-years.

At baseline, patients were classified on average as overweight (BMI ≈ 25 kg/m^2^), with higher systolic blood pressure (mean SBP ≥ 120 mmHg) and with higher fasting glucose levels classified in the prediabetes level (mean fasting glucose = 121.47 mg/dL). Regarding total cholesterol, 44% of the subjects were characterized by altered values (total cholesterol ≥200) and 51% had higher diastolic blood pressure (DBP ≥ 80 mmHg). Additionally, 37% of the patients with high diabetes risk indicated family history of diabetes during enrollment, 14% were current smokers, 28% were alcohol drinkers, and 32% engaged in regular physical activity (Table 1).

### Survival analysis

Analyzing the results of the univariate models, we found that age, BMI, SBP, ALT, total cholesterol, and family history of diabetes were significant variables at α = 5% (Table 2). When these variables were considered in multivariate analysis using the forward stepwise procedure, BMI, age, total cholesterol, and family history of diabetes remained significant (Table 2). Subjects with higher diabetes risk were older (age ≥ 53; hazard ratio [HR] = 1.250; 95% CI = 1.086, 1.439; Table 3), with higher BMI (BMI ≥ 25; HR = 1.356; 95% CI = 1.133, 1.625; Table 3) and total cholesterol (total cholesterol ≥ 200 mg/dL; HR = 1.219; 95% CI = 1.058, 1.405; Table 2). Also, subjects with a family history of diabetes had a 58% higher risk of diabetes than those with no family history of diabetes (HR = 1.58; 95% CI = 1.368, 1.817; Table 2). Analyzing the incidence of diabetes in each category of the significant variables, we found 230 cases per 1,000 person-years when BMI was greater than 25. In subjects ≥ 53 years of age, 231 cases per 1,000 person-years were found. There were 235 and 263 cases per 1,000 person-years for total cholesterol ≥200 and family history of diabetes, respectively (Supplementary Table 1).

**Table 2:** Results of univariate and multivariate Cox proportional hazards models.

Variables	HR [95% CI]		*P*-value	
**Univariate Cox models**				
Age (≥53)	1.274 [1.108, 1.465]		<0.001	
BMI (Level 1)	1.295 [1.046, 1.603]		0.017	
BMI (Level 2)	1.462 [1.225, 1.746]		<0.001	
SBP (Level 1)	—		No sig.	
SBP (Level 2)	1.407 [1.152, 1.717]		<0.001	
DBP (≥80)	—		No sig.	
AST (>40)	—		No sig.	
ALT (>40)	1.243 [1.054,1.466]		0.009	
Total cholesterol (≥200)	1.308 [1.138, 1.504]		<0.001	
Family history of diabetes (yes)	1.565 [1.358, 1.802]		<0.001	
Gender (Male)	—		No sig.	
Current smoker (yes)	—		No sig.	
Alcohol drinker (yes)	—		No sig.	
Physical activity (yes)	—		No sig.	
**Multivariate Cox model**				
**Variables**	**β (standard error)**	**HR [95% CI]**	***P*** **-value**	Score*
Age (≥53)	0.223 (0.072)	1.250 [1.086, 1.439]	0.002	2
Family history of diabetes (yes)	0.455 (0.073)	1.576 [1.368, 1.817]	<0.001	5
BMI (Level 1)	0.242 (0.109)	1.274 [1.028, 1.578]	0.0270	2
BMI (Level 2)	0.305 (0.092)	1.356 [1.133, 1.625]	<0.001	3
Total cholesterol (≥200)	0.198 (0.072)	1.219 [1.058, 1.405]	0.006	2

^*^Each score was computed by dividing the value of each coefficient by the minimum value (β_total cholesterol_ = 0.198) and multiplying it by a constant (k = 2).

**Table 3:** Multivariate model of the KARE cohort.

Variables	β (standard error)	HR [95% CI]	*P*-value	Score
Age (>53)	0.1535 (0.069)	1.166 [1.018, 1.335]	0.0261	2
BMI (≥25)	0.2639 (0.069)	1.302 [1.138, 1.489]	<0.001	3
SBP (≥120)	0.2620 (0.069)	1.300 [1.135;1.488]	<0.001	3
Family history of diabetes (yes)	0.3504 (0.081)	1.420 [1.211, 1.664]	<0.001	5

^*^We corrected for the effects of gender, maintaining it in the model. Also, we included high-density lipoprotein cholesterol (HDL) and triglycerides, which were significant in the multivariate model, but we did not compute a score for them because they were not present in the KNDP cohort (many missing values for these variables).

The English in this document has been checked by at least two professional editors, both native speakers of English. For a certificate, please see: http://www.textcheck.com/certificate/YetViu.

### Internal validation via bootstrapping

**Figure 2:**
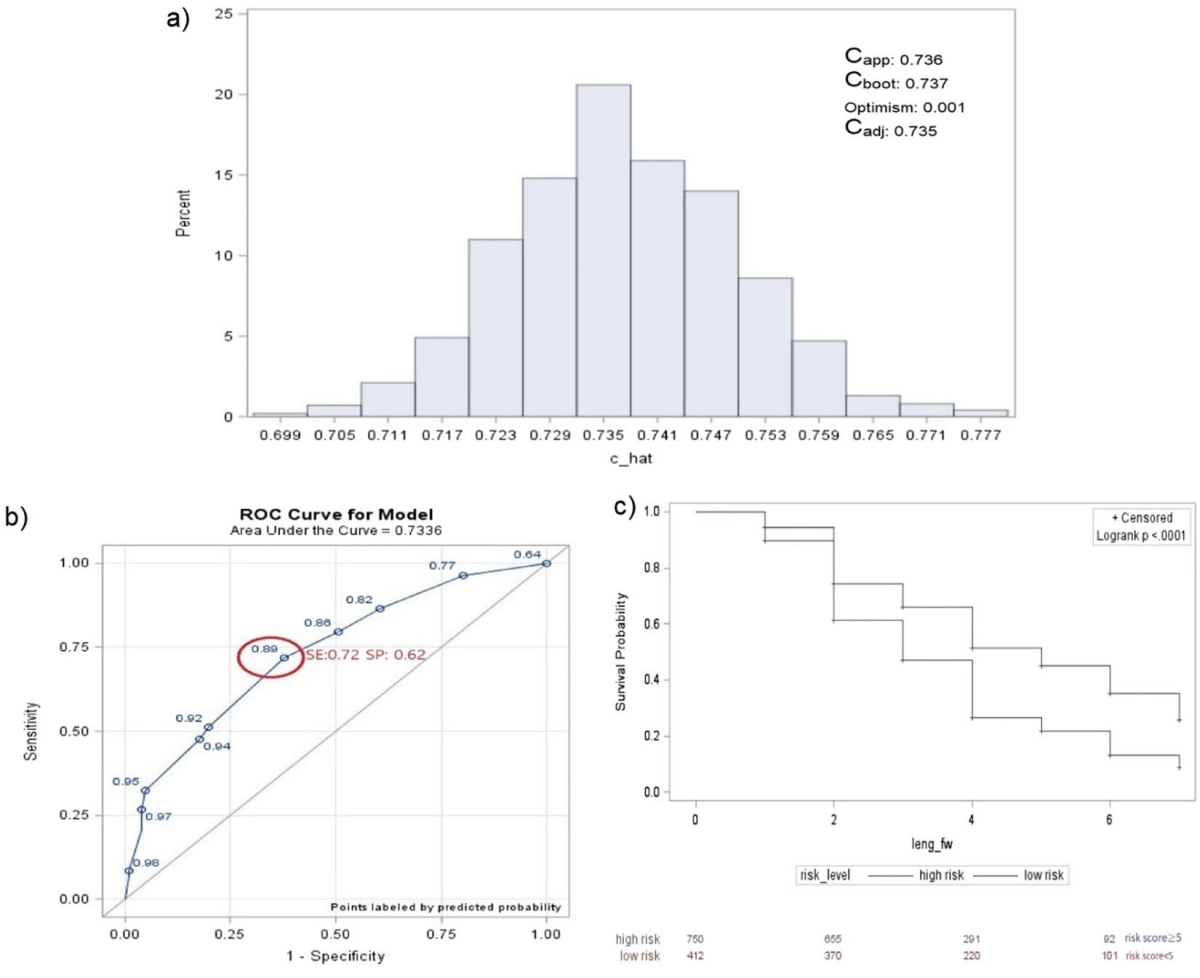
(**a**) C-index distribution of 1,000 bootstrap samplings. (**b**) Receiver operating characteristic (ROC) curve analysis using risk scores. (**c**) Comparison of survival estimates between the high- and low-risk groups based on risk scores. ^*^(**a**) C_app_ = c-index of the developed model (apparent discrimination); C_boot_ = c-index of the developed models of the 1,000 bootstrap samples; C_adj_ = C_app_ − optimism (adjusted c-index). ^**^(**a**) The range of C_boot_ that remained larger than 0.70 showed a good performance in the validation step. ^*^(**b**,**c**) Better threshold: patients with a higher risk of diabetes = score ≥ 5; patients with lower risk of diabetes = score < 5.

The developed model showed good performance (C_app_ = 0.736, Fig. 2a) and the c-index computed on the bootstrap samples showed a similar value (C_boot_ = 0.737, Fig. 2a) with a range of values that remained larger than 0.70 (0.70–0.78, Fig. 2a). The good performance of our model persisted after correction for optimism (C_adj_ = 0.735, optimism = 0.001, Fig. 2a), showing that the variables selected through our algorithm predicted diabetes risk.

### Risk score development

A diabetes risk score was computed (ranging from 0 to 12) as the sum of the scores given by the coefficients estimated in the final validated model. Subjects with the highest risk score (score =12) were ≥ 53 years of age, with a family history of diabetes, BMI ≥ 25, and total cholesterol level ≥200. In order to define a cut-off that best discriminates between patients with high and low diabetes risks, the values of sensitivity (SE) and specificity (SP) were computed when varying the threshold. A better threshold was defined by a score ≥ 5 (SE = 72% and SP = 62%, AUC = 0.73; Fig. 2b) with SE ranging from 0.69 to 0.74 and SP from 0.55 to 0.69 from the bootstrapped samples.

**Figure 3:**
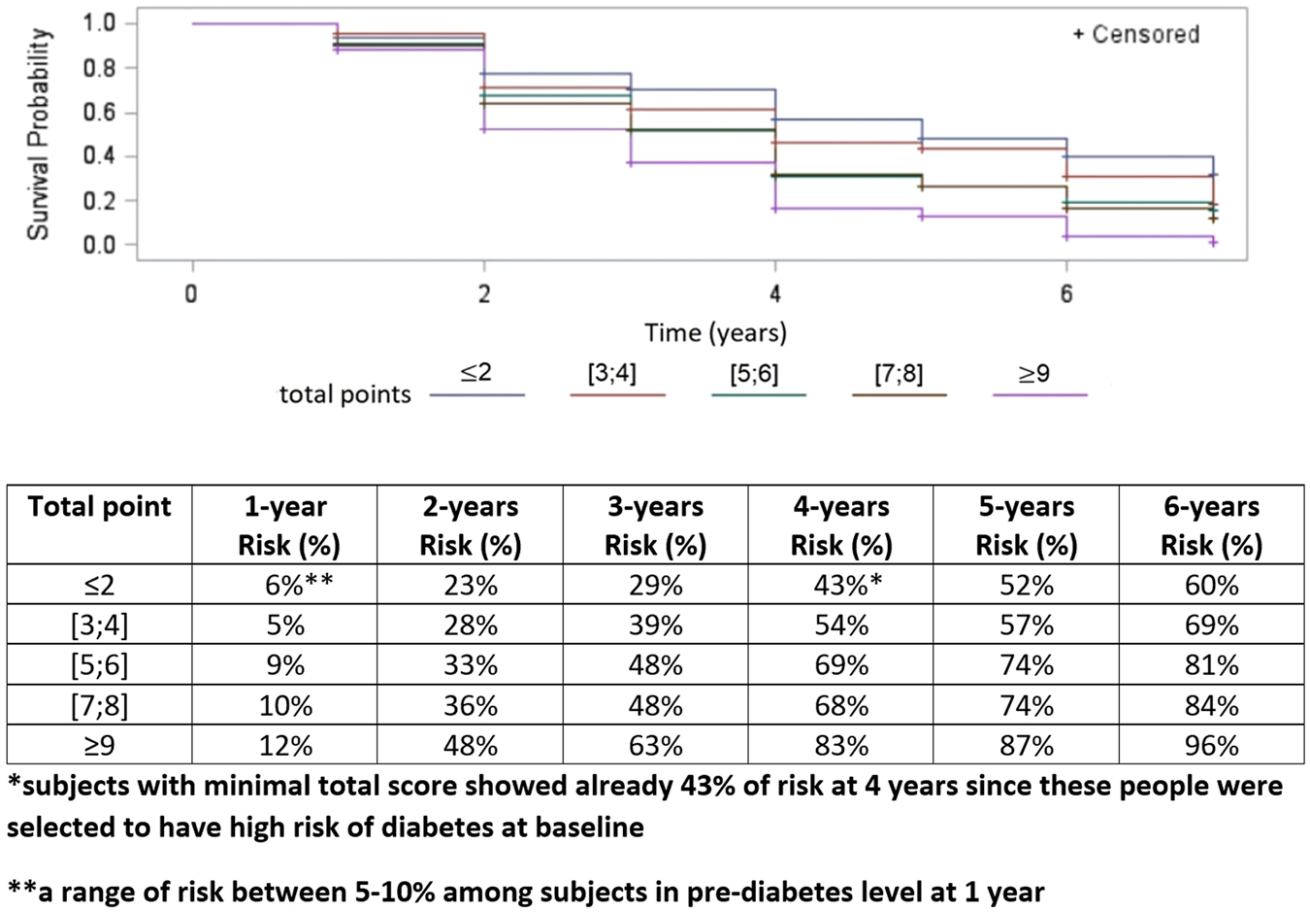
Diabetes risk distribution over time based on total risk scores among prediabetic subjects. *The total points obtained by our risk score system ranged from 0 to 12. We grouped the total points in classes to obtain reasonable risk values, because of the presence of small sample sizes for some total scores.

People with a high diabetes risk score (risk score ≥ 5) were represented by a lower survival curve than the group with low risk, showing a significantly higher diabetes risk than the other set of subjects (log-rank *P*-value < 0.001; Fig. 2c). Analyzing the total points (Fig. 3) developed with this risk score system, we noticed that subjects who had scores ≤ 2 were already characterized by a high risk (43%) within 4 years, due to the presence of subjects at baseline at high risk of diabetes.

According to the literature^1^, pre-diabetic subjects showed a diabetes risk of 5–10% at 1 year (1-year risk = 6%, Fig. 3). Increasing the total score, the risk tended to increase over time, and a total point value of 3–4 led to a risk of over 50% within 4 years. Also, subjects with the highest point total (≥9) showed a very high risk of diabetes at 6 years, equal to 96%.

### Comparison with external cohort

Subjects in the KARE cohort were followed for 10 years (2002–2012), and 30% of them presented with diabetes during a mean follow-up of 8.43 ± 0.05 years. The incidence of diabetes was 42 cases per 1,000 person-years. At baseline, they were classified on average as overweight (BMI ≈ 25 kg/m^2^), with higher systolic blood pressure (mean SBP ≥120 mmHg; Supplementary Table 2). Among them, 17% indicated a family history of diabetes, 26% were current smokers, and 47% were current alcohol drinkers (Supplementary Table 2). Analyzing the multivariate model, we obtained age, BMI, SBP, and family history of diabetes as significant variables (Table 3). When we determined a score for each significant variable using the same method applied to the KNDP variables, we confirmed that family history of diabetes produced the highest score (score = 5), as in the KNDP cohort (Table 3). Additionally, we found a score ≥5 as the best cut-off that correctly classified subjects by diabetes status (SE = 69%, SP = 45%).

## Discussion

Many subjects with high diabetes risk presented with the disease (69%) after 4 years on average, particularly people who were older with a family history of diabetes, higher BMI, and higher total of cholesterol. Using our predictive model, validated by the bootstrap method, we defined a diabetes risk score that correctly classified 72% of patients with diabetes and 62% without the disease, defining a cut-off equal to 5. Using this risk score system, people with high diabetes risk presented with the disease with greater probability if they had only a family history of diabetes (risk score = 5). This result was also confirmed when we analyzed an external cohort with the same procedure: the same score was given for BMI, age, and family history of diabetes. In particular, the same threshold (score ≥5) divided subjects with higher and lower risk of diabetes, confirming that a family history of diabetes in a prediabetic subject was enough to classify the subject with higher risk, regardless of other clinical characteristics.

Comparing our study with a previous one that developed a diabetes risk score for the Korean population^14^, we found a large discrepancy related to the score assigned for the presence of a family history of diabetes. In our system, we assigned a score that was five times higher than that given by the other Korean risk score system. Our risk score system was more similar to one developed in a rural Chinese population^11^ that assigned a higher score to a family history of diabetes variable. The latter study analyzed a prospective cohort using our statistical methodology to implement a predictive model, suggesting that the differences between our score system and the other applied to a Korean population could be due to distinctly different analytical approaches. Indeed, in the case of long follow-up periods, Cox proportional hazards models show more statistical power than logistic regression models^21,22^.

Also, in contrast to the diabetes risk score already established for the Korean population, our risk score is reliable only in subjects with higher risk (prediabetic level) and cannot be applied to the entire Korean population, as it is based on a sensitive subgroup of people.

In conclusion, we confirmed the importance of family history of diabetes in assessing diabetes risk by also analyzing an external cohort using the same methodology. This result was even stronger, as the distribution of the clinical characteristics at baseline and the distribution of the events was dissimilar between the two cohorts, confirming the possibility for extending this result to the general population.

Unfortunately, not all the significant variables were in accordance between the two cohorts. SBP was significantly predictive of diabetes in the KARE cohort, but not in the KNDP cohort; instead, there was only a small effect of total cholesterol on diabetes risk. This discrepancy was likely a result of not all subjects in the KNDP cohort being selected based on glucose levels at baseline, as was the case in the KARE cohort. Indeed, one group of subjects in the KNDP cohort was selected with high risk at baseline because they had diabetes during cohort enrollment. At any rate, a strong effect of family history of diabetes was confirmed for prediabetic subjects, and it seems that the transition from a prediabetic condition to diabetes was strongly related to the presence of a family history of diabetes, regardless of the other clinical values. Because family history of diabetes seems to be affected not only by genetic patterns but also by environmental factors (e.g., subjects in the same family often share similar environments^23^), it will be important in future studies to analyze the effects of gene–environment interactions on diabetes risk.

## Electronic supplementary material

Supplementary information

## Data Availability

The datasets generated in the current study are available from the corresponding authors upon reasonable request.
