# Supplementary material for: Importance of family history of diabetes in computing a diabetes risk score in Korean prediabetic population
Source: Sci Rep. 2018 Oct 29;8:15958. doi: 10.1038/s41598-018-34411-w (PMC6206127; doi:10.1038/s41598-018-34411-w)
Supplement: Supplementary file 1 — Supplementary information [file 41598_2018_34411_MOESM1_ESM.docx]

**Importance of family history of diabetes in computing a diabetes risk score in Korean prediabetic population**

Morena Ustulin^1^^¶^, Sang Youl Rhee^2¶^, Suk Chon^2^, Kyu Keung Ahn^2^, Ji Eun Lim^3^, Bermseok Oh^3^, Sung-Hoon Kim^4^, Sei Hyun Baik^5^, Yongsoo Park^6^, Moon Suk Nam^7^, Kwan Woo Lee^8^,

Young Seol Kim^2^, and Jeong-Taek Woo^2^

Supplementary Table 1: Diabetes mellitus incidence in the Korea National Diabetes Program (KNDP) cohort.

| Variables | N (%) | Total  person-years | Mean person-years  [95% Cl] | DM Incidence  (cases per 1,000 person-years) |
| --- | --- | --- | --- | --- |
| Age (≥ 53) | 539 (46%) | 1704 | 3.16  [3.03, 3.29] | 231 |
| Gender (Males) | 560 (48%) | 1826 | 3.26  [3.13, 3.40] | 235 |
| BMI (Level 1) | 253 (22%) | 840 | 3.32  [3.12, 3.52] | 205 |
| BMI (Level 2) | 607 (52%) | 1987 | 3.27  [3.14, 3.41] | 230 |
| SBP (Level 1) | 565 (49%) | 1900 | 3.36  [3.22, 3.50] | 207 |
| SBP (Level 2) | 210 (18%) | 652 | 3.10  [2.89, 3.32] | 248 |
| DBP (≥ 80) | 598 (51%) | 1991 | 3.33  [3.19, 3.47] | 218 |
| ALT (> 40) | 217 (19%) | 715 | 3.29  [3.09, 3.50] | 241 |
| AST (> 40) | 111 (10%) | 352 | 3.17  [2.89, 3.45] | 241 |
| Total cholesterol (≥ 200) | 516 (44%) | 1671 | 3.24  [3.10, 3.38] | 235 |
| Family history of diabetes (yes) | 428 (37%) | 1276 | 2.98  [2.83, 3.13] | 263 |
| Current smoker  (yes) | 161 (14%) | 543 | 3.37  [3.12, 3.63] | 241 |
| Alcohol drinker (yes) | 322 (28%) | 1101 | 3.42  [3.23, 3.61] | 228 |
| Physical activity (yes) | 370 (32%) | 1216 | 3.29  [3.12, 3.45] | 254 |

Supplementary Table 2. Characteristics of the Korea Association Resource (KARE) cohort at baseline.

| 3,151 subjects observed from 2002–2012 | |
| --- | --- |
| Variable | n (%) or mean [95% CI] |
| Age (years) | 53.18 [52.87, 53.49] |
| BMI (kg/m^2^) | 25 [24.76, 24.98] |
| Gender (Males) | 1506 (48%) |
| SBP (mmHg) | 122.05 [121.39, 122.72] |
| DBP (mmHg) | 80.47 [80.05, 80.88] |
| Total cholesterol (mg/dL) | 195.65 [194.44,196.86] |
| AST (IU/L) | 29.94 [29.35, 30.52] |
| ALT (IU/L) | 28.72 [27.93, 29.51] |
| HDL | 44.51 [44.15, 44.86] |
| Triglycerides | 169.31 [165.64, 172.98] |
| Family history of diabetes (yes) | 527 (17%) |
| Non-smoker | 1827 (58%) |
| Former smoker | 516 (16%) |
| Current smoker | 808 (26%) |
| Non-drinker | 1458 (46%) |
| Former drinker | 213 (7%) |
| Current drinker | 1480 (47%) |
